# Easy‐to‐use $B_{1}^{+}$ shims for human brain imaging at 7 T

**DOI:** 10.1002/mrm.30617

**Published:** 2025-06-24

**Authors:** Emma J. P. Brouwer, Wietske van der Zwaag, Jurjen Heij, Nikos Priovoulos

**Affiliations:** ^1^ Spinoza Centre for Neuroimaging Netherlands Institute for Neuroscience Amsterdam the Netherlands; ^2^ Computational Cognitive Neuroscience and Neuroimaging Netherlands Institute for Neuroscience Amsterdam the Netherlands; ^3^ Department of Biomedical Engineering and Physics Amsterdam UMC Amsterdam the Netherlands; ^4^ Oxford Centre for Integrative Neuroimaging FMRIB, Nuffield Department of Clinical Neurosciences, University of Oxford Oxford UK

**Keywords:** 7 T, $B_{1}^{+}$ shimming, calibration‐free, fMRI, high field, phase shimming

## Abstract

**Purpose:**

$B_{1}^{+}$ field inhomogeneity is a common problem in high field brain MRI (>3 T). Parallel‐transmit methods that adjust the $B_{1}^{+}$ field channelwise often require valuable scan time. Group‐optimized phase shims are presented to increase or attenuate the $B_{1}^{+}$ field in specific brain regions, omitting personalized calibrations and potentially enabling reduced FOV acquisitions or artifact reduction.

**Methods:**

Channelwise $B_{1}^{+}$ maps were obtained for seven participants using an 8Tx/32Rx coil and a 7 T MRI scanner. Two regional shim settings ($B_{1}^{+}$ shims) were calculated: one to increase the $B_{1}^{+}$ field in the cerebellum and the other to increase the $B_{1}^{+}$ field in the occipital lobe while attenuating the $B_{1}^{+}$ field in the frontal lobe. $B_{1}^{+}$ maps from five participants outside the design group were used to simulate the $B_{1}^{+}$ profiles, and seven were scanned to evaluate the implementation of the $B_{1}^{+}$ shims using $B_{1}^{+}$ maps, 3D EPI, GRE acquisitions, and a visual fMRI experiment.

**Results:**

Both regional shim settings successfully amplified the $B_{1}^{+}$ field in the selected ROIs resulting in improved $B_{1}^{+}$ yield and increased tSNR in the 3D EPI images and fMRI experiments compared to the circularly polarized shim mode. The attenuating $B_{1}^{+}$ shim decreased $B_{1}^{+}$ in the frontal ROI, decreasing fold‐over artifacts in a reduced FOV, lowering g‐factors in accelerated scans with high undersampling factors and resulted in improved BOLD responses in the visual fMRI experiment.

**Conclusion:**

Regional $B_{1}^{+}$ shim settings remove the need for time‐consuming, personalized $B_{1}^{+}$ measurements and calibrations. The attenuating shim allows for signal reduction within the power limits of the rf‐coil, reducing artifacts while improving the $B_{1}^{+}$ field in selected ROIs.

## 1 INTRODUCTION

High‐field MRI (>3 T) has transformed the field of neuroscientific research by providing increased signal‐to‐noise ratio (SNR) and contrast‐to‐noise ratio (CNR) compared to lower field strengths.^1^, ^2^ In functional MRI research, the increased SNR is often traded for increased spatial and/or temporal resolution. Commonly, instead of studying the whole brain, high‐resolution fMRI is used to investigate function of a specific part of the brain, such as the occipital lobe,^3^ the cerebellum^4^ or the motor cortex.^5^ Through focusing on these structures specifically, functional subunits of brain regions, such as cortical columns and layers, have been studied.^6^, ^7^, ^8^

**FIGURE 1:**
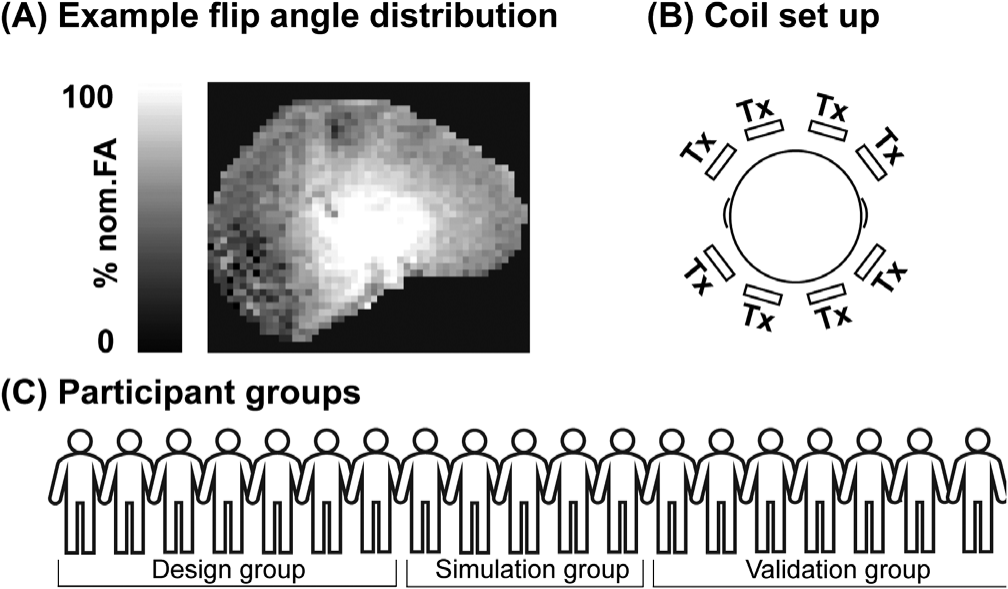
(A) An example of a flip angle distribution in pseudo‐CP mode. (B) A schematic overview of the coil elements of the 8Tx/32Rx Nova coil. (C) An overview of the participants in each group: Data from group 1 (N=7) were used to design regional shim settings 1 and 2 (RSS‐1, RSS‐2). Data from group 2 (N=5) were used to simulate the implementation of RSS‐1 and RSS‐2 outside the design group. Data from group 3 (N=7) were used to acquire new data showcasing the implementation of RSS‐1 and RSS‐2.

High‐field MRI suffers from $B_{1}^{+}$ field inhomogeneities.^1^, ^9^, ^10^ The RF wavelength is inversely proportional to the static magnetic field strength and becomes comparable to the human head size at 7 T.^11^, ^12^ A shorter RF wavelength results in destructive and constructive interferences of the $B_{1}^{+}$ field. Within the human head, using commonly available transmit coil geometries, areas such as the cerebellum are most affected by these inhomogeneities (Figure 1A).^13^, ^14^ This results in reduced SNR and CNR in these regions. This is unfortunate because such regions can be the ones that precisely benefit most from high‐field imaging; for example, the cerebellum has a densely foliated structure which benefits greatly from imaging with high spatial resolution.^4^, ^15^, ^16^, ^17^ In order to reduce $B_{1}^{+}$ field inhomogeneities in the cerebellum, altered hardware set‐ups have been proposed such as dielectric pads and alternative coil designs.^18^, ^19^, ^20^ While these techniques can partially enhance the $B_{1}^{+}$ field, they offer limited flexibility.

Parallel transmit (pTx) coils can be used to reduce $B_{1}^{+}$ field inhomogeneities.^21^, ^22^, ^23^ PTx coils consist of multiple transmit elements with which the $B_{1}^{+}$‐field can be “shimmed”. PTx systems can be used to improve the resulting excitation either statically^10^, ^24^, ^25^, ^26^, ^27^ that is, with a combination of channelwise phase offsets and magnitude differences between channels that remain constant throughout the excitation, or dynamically,^23^, ^28^, ^29^, ^30^, ^31^ that is, with a further manipulation of the RF (sub)pulses in the temporal domain to result in a homogeneous excitation. However, calculating such a $B_{1}^{+}$ shim on an individual level requires $B_{1}^{+}$ maps to be acquired and the subsequent shim or pulse calculation makes such pTx approaches relatively time‐inefficient.^18^, ^21^ Unfortunately, this additional acquisition and analysis time limits neuroscientific or clinical application.

To reduce this individual level calibration, universal precalculated RF waveforms have been developed taking advantage of the relatively consistent geometry of the human head.^22^ Universal pulses show little reduction in the performance compared to the individually optimized cases and find their way into regions beyond the brain^32^ as well as clinical settings.^33^ However, the universal dynamic pTx pulse waveforms remain complex compared to the static pTx implementations, increasing computational demands on the waveform generator and potentially slowing down the acquisition. Dynamic pTx pulses also tend to be longer than standard pulses, potentially impacting TE and TR. Additionally, slab‐selective pTx pulses lead to higher SAR and peak power demands than nonselective pTx pulses.

An alternative approach is to explore the generalizability of the static RF shims, given the relatively consistent head geometry. Recently, this approach was shown to be efficient in the spinal cord^34^ and for single voxel MRS in small regions of interest in the brain.^35^ Neuroscientific studies using field strengths above 3 T frequently suffer from artifacts arising from areas of high susceptibility as well as undersampling‐related aliasing artifacts. Image reconstruction at high acceleration factors can be improved by attenuating the signal of unwanted regions, this can be done by modifying the slice or volume excitation. This can be done using selective‐excitations^36^, ^37^ or reduced FOV acquisitions with outer‐volume suppression which come at the cost of increased SAR deposition.^38^, ^39^, ^40^ Selective excitations have been presented in combination with pTx,^32^, ^41^ facilitating an increase in acceleration factors in BOLD fMRI experiments. These approaches can be particularly useful to improve scan quality in neuroscientific research. Nevertheless, pTx solutions have to adhere to stricter power limits, they are often vendor specific and frequently require individual optimizations.

In the current study, we explore the implementation of group optimized attenuating and increasing $B_{1}^{+}$ shims in selected regions of interest in the brain using a 8Tx/32Rx coil at 7 T allowing reduced FOV acquisitions as well as reduced g‐factors at higher acceleration factors.

## 2 METHODS

### 2.1 Participants

Data from 20 participants (age, 20–44, 11 Female) were included in this study. In accordance with the declaration of Helsinki, all participants provided written informed consent and the study was approved by the local ethics committee. The participants were divided into three groups (Figure 1C). We used $B_{1}^{+}$ maps of participants from group 1 (n=7) to calculate phase offsets that optimized the $B_{1}^{+}$ field in a selected ROI. We simulated the generalizability of these using an independent group of $B_{1}^{+}$ maps collected from group 2 (n=5). We finally validated these results in group 3 (n=8) across several acquisitions.

### 2.2 Data acquisition

Participants were scanned using a Philips Achieva 7T MRI equipped with an 8Tx/32Rx whole‐head coil (Nova Medical, USA). The details for all acquisitions are summarized in Table 1. For participants in groups 1 and 2, DREAM $B_{1}^{+}$ maps^42^ were acquired in the pseudo‐circularly polarized (pseudo‐CP) mode as well as spoiled gradient echo's (SGEs) for each transmit channel (n=8). These were used to calculate (group 1) and simulate (group 2) the transmit phase offsets for two different regional $B_{1}^{+}$ shim settings:

1. **Regional Shim Setting 1 (RSS‐1):** A region selective increasing $B_{1}^{+}$ field shim for the cerebellum. Seven transmit phase offsets were calculated by optimizing the $B_{1}^{+}$ field in a cerebellar ROI in group 1. The cerebellar ROIs were manually created in FSL for each participant in group 1. The average ROI size of the cerebellar ROI mask was $126{\text{cm}}^{3}(\text{SD}=27{\text{cm}}^{3})$.
2. **Regional Shim Setting 2 (RSS‐2):** A region selective increasing‐and‐attenuating $B_{1}^{+}$ field shim. Seven transmit phase offsets were calculated by maximizing the $B_{1}^{+}$ field in the occipital lobe while attenuating the $B_{1}^{+}$ field in the frontal lobe of the brain (to reduce fold‐over artifacts). The masks for the occipital lobe and frontal lobe were manually created in FSL for each participant in group 1. The average ROI mask size was 91 ${\text{cm}}^{3}$ (SD = 21 ${\text{cm}}^{3}$) in the occipital lobe and 140 ${\text{cm}}^{3}$ (SD = 32${\text{cm}}^{3}$) in the frontal lobe, see Supporting Information Table 1 for all respective ROI sizes per participant. Note that we opted for an occipital lobe ROI versus a frontal lobe ROI to showcase the increasing‐and‐attenuating shim, rather than a cerebellar ROI, to best profit from the existing coil layout.^25^ See Supporting Information Figure 2 and Tables 2 and 3 for the effect of the ROI size on the resulting FA% in the frontal lobe and occipital ROIs.

**TABLE 1:** Acquisition parameters.

Acquisition	FOV	Res (mm)	FA	TE (ms)	TR (ms)	TRvol	Tacq (min)	PF (y/z)	Group	$B_{1}$+ Shim
DREAM $B_{1}$+ map	224×224×168	3.5×3.5×3.5	7	0.9	20	‐	01:03	‐	1, 2, 3	pCP, RSS‐1, RSS‐2
SGE for each Tx	192×60×192	3.5×3.5×3.5	1.5	2	8	‐	03:12	‐	1,2	pCP, RSS‐1, RSS‐2
3D EPI full FOV	186×186×120	2.0×2.4×2.0	20	21	69	8.5	06:32	0.80/0.80	3	pCP, RSS‐1, RSS‐2
3D EPI reduced FOV	186×89×120	2.0×2.4×2.0	20	21	69	4.2	03:17	0.80/0.80	3	pCP, RSS‐2
3D (GRE) full FOV	220×220×164	2.0×2.0×2.0	7	8	18	‐	02:40	0.75/0.75	3	pCP, RSS‐1, RSS‐2
3D (GRE) reduced FOV	246×103×174	2.0×2.0×2.0	7	8	18	‐	01:23	0.75/0.75	3	pCP, RSS‐2
EPI during task reduced FOV	186×89×120	2.0×2.4×2.0	20	21	69	8.5	06:32	0.80/0.80	3 (*n* = 1)	pCP, RSS‐2
EPI during task full FOV	186×186×120	2.0×2.4×2.0	20	21	69	8.5	06:32	0.80/0.80	3 (n=1)	pCP, RSS‐2

*Note*: The parameters of the obtained acquisitions, for which group they were obtained and the shim setting that was applied: pCP, pseudo‐Circularly Polarized mode; PF, partial Fourier; RSS‐1, Regional Shim Setting 1; RSS‐2, Regional Shim Setting 2; FOV, field of view; Res, Resolution; SGE, spoiled gradient echo.

RSS‐1 and RSS‐2 were validated in group 3: DREAM $B_{1}^{+}$ maps, slab‐selective 3D‐EPI (Echo Planar Imaging), and GRE (Gradient Echo) data were acquired using RSS‐1, RSS‐2, and using the pseudo‐CP mode (Table 1). 3D EPI acquisitions were performed to assess BOLD signal stability over time and fold‐over artifacts in the posterior–anterior direction of the transverse slab. GRE acquisitions were performed to assess fold‐over artifacts in the posterior–anterior direction and for registration purposes. DREAM $B_{1}^{+}$ maps were acquired to assess percentage nominal flip angle (FA) distributions. To demonstrate the feasibility of increasing the undersampling factor when using RSS‐2, for one participant, vendor generated g‐factor maps were obtained across seven acceleration factors (SENSE 2‐8) during a whole brain 3D EPI acquisition. Local SNR improves when g‐factors are low.^43^

To demonstrate the usage of these shim settings in an fMRI experiment, for one participant in group 3, 3D EPI acquisitions using RSS‐2 and pseudo‐CP mode were performed during a 6 min 30 s block design visual task consisting of an 8 Hz flickering checkerboard presented on the entire screen for 30 s off/on. These acquisitions were performed with reduced (90 volumes) and full (45 volumes) FOV, where both had a $T_{acq}$ of 6 min 32 s. Note that, in the fMRI experiment, to reduce interactions between the $B_{1}^{+}$ shims and the reconstruction, we did not use GRAPPA or SENSE undersampling for the 3D EPI and GRE acquisitions. This came at a cost of longer echo trains, in the 3D EPI images, but allowed us to isolate the effect of the $B_{1}^{+}$ shims.^1^

### 2.3 Cost functions and regional shim setting calculations

The $B_{1}^{+}$ shims were optimized in the following manner: For the case of a pTx system with eight transmits, the $B_{1}^{+}$ field for a given voxel is given by

(1) $$B_{1\text{shimmed}}=\overset{t=8}{\underset{t=1}{\sum }}B_{1unshimmed}\times e^{i{\bar{x}}_{t}},$$

where ${\bar{x}}_{t}(-\pi <x_{t}<\pi )$ is the phase offset of each transmit channel t. To optimize the $B_{1}^{+}$ field over the whole group, we used a min–max approach, similar to previous studies.^22^, ^35^ Alternatively, calculating the median across the group can also be used.^45^ For ${\bar{x}}_{t}$ over n = 7 participants in group 1, we are trying to minimize a cost function g(x) as follows:

(2) $$Min(Max\left(\overset{n=7}{\underset{n=1}{\sum }}g({\bar{x}}_{t})\right).$$

With g(x) as the cost function of the $B_{1}^{+}$ over the given ROI. g(x) can be designed accordingly to the desired optimization. To solve the min–max problem, we employed MATLAB's fmincon function using the Interior Point algorithm (Optimization Toolbox). To calculate the transmit phase offsets that increase the $B_{1}^{+}$ field over a given ROI, we used the following cost function $g_{1}(x)$:

(3) $$g_{1}({\bar{x}}_{t})=\frac{\sigma_{B_{1shimmed}}}{{\bar{B}}_{1shimmed}^{2}},$$

where $\sigma_{B_{1shimmed}}$ is the standard deviation of the $B_{1}^{+}$ field, and ${\bar{B}}_{1shimmed}^{2}$ is the $B_{1}^{+}$ field squared in the target ROI. To calculate the eight resulting transmit phase offsets for RSS‐1, we minimized Equation 3 using Equation 2 where the target ROI is the cerebellum.

In order to attenuate the $B_{1}^{+}$ field over a given ROI, we used $g_{2}(x)$:

(4) $$g_{2}({\bar{x}}_{t})={\bar{B}}_{1shimmed}\times \sigma_{B_{1shimmed}},$$

where ${\bar{B}}_{1shimmed}$ denotes the mean $B_{1}^{+}$ over a given ROI^2^.

To calculate the eight transmit phase offsets for RSS‐2, we created a combined cost function using Equation 3 (increasing) where the target ROI is the occipital lobe as well as Equation 4 (attenuating), where the target ROI was the frontal lobe. The combined and final cost function for the RSS‐2 was defined as

(5) $$g_{3}({\bar{x}}_{t})=g_{1}(x)\times g_{2}{\left(x\right)}^{2}$$

In equation 5, $g_{2}(x)$ is squared because earlier simulations showed that, given our head coil and field strength, this led to a solution that increased the performance of the destructive component without compromising the constructive ROI and resulted in more consistent FA percentages across participants in the frontal lobe within group 1. Pilot experiments^46^ showed that using only equation (3) as a cost function led to signal loss outside of the attenuating target ROI.

Both RSS‐1 and RSS‐2 can be optimized personally for a specific participant by adjusting n=1 in Equation 2 or across a group by adjusting $n=n_{group}$.

### 2.4 Data analysis

#### 2.4.1 Regional shim setting 1

Nominal FA percentages were calculated from DREAM $B_{1}^{+}$ acquisitions according to Nehrke et al.^42^ To illustrate that RSS‐1 could be used in a calibration free manner (i.e., in participants outside the design group [group 1]), nominal FA percentages were compared between groups 1 and 2 in the cerebellum. All comparisons were done using a paired t‐test (MATLAB) and by visual inspection of the images in FSLeyes. To illustrate variability within groups, nominal FA percentages were compared between the group optimized and personally optimized RSS‐1 in groups 1 and 2. To validate the $B_{1}^{+}$ shims in real‐life settings, we further used them in group 3, where the percentage of the nominal FA and tSNR were compared between the pseudo‐CP mode and RSS‐1 in an ROI within the cerebellum (15×15×15 voxels placed in the right posterior lobe of the cerebellum).

#### 2.4.2 Regional shim setting 2

Using the occipital lobe ROI and the frontal lobe ROI masks, nominal FA percentages were compared between RSS‐2 and the pseudo‐CP mode in these regions. 3D EPI data acquired with RSS‐2 and in pseudo‐CP mode and in both large and reduced FOV acquisitions were visually evaluated for fold‐over artifacts and image quality. From these 3D EPI acquisitions, the tSNR was calculated in an ROI in the occipital lobe (15×15×15 voxels placed in V1).G‐factor maps were compared in a occipital lobe ROI across seven acceleration factors using Matlab. To assess the differences between g‐factors between the pseudo‐CP shim and RSS‐2, voxel‐wise difference images ((pseudo‐CP)‐(RSS‐2)) were calculated for each acceleration factor.

FMRI data were motion corrected using McFlirt^47^ and analyzed using a FSL FEAT first‐level GLM. The resulting Z scores were compared between the large FOV and the reduced FOV and between the pseudo‐CP mode and RSS‐2.

## 3 RESULTS

### 3.1 Regional shim setting 1

**FIGURE 2:**
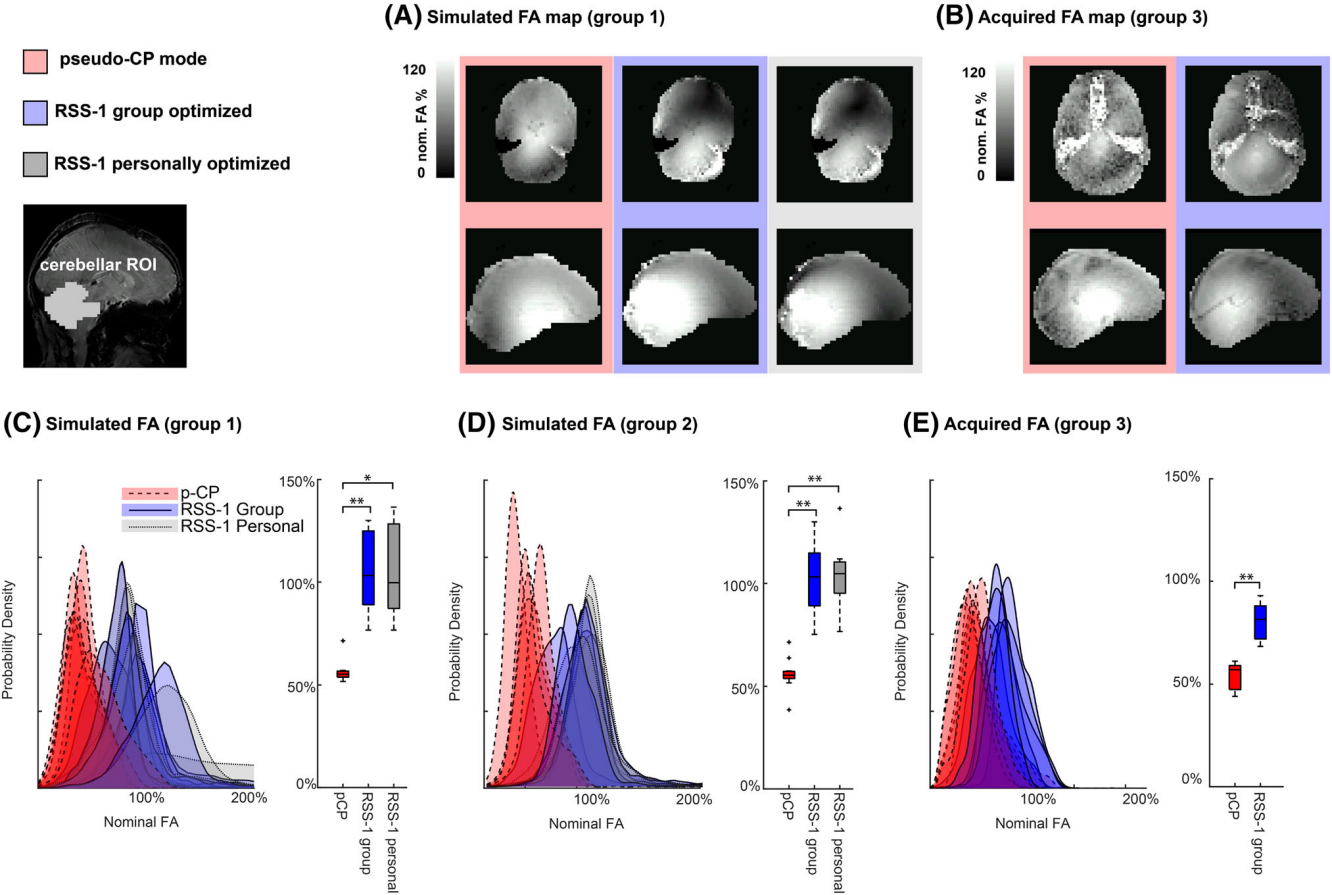
(A) The simulated FA map for a participant in group 1. (B) The acquired FA map for a participant in group 3. (C–E) Nominal percent FA distributions and group averaged box plots in the cerebellum for the pseudo‐CP mode (red), RSS‐1 optimized for each participant personally (gray) and RSS‐1 optimized over group 1 (blue) in group 1 (C), group 2 (D), and group 3 (E). The similarity between the FA distributions in the personalized and group optimized RSS‐1. pCP, pseudo‐circularly polarized; FA, flip angle, nom. FA % = nominal percent flip angle. *p<0.05, **p<0.01.

**FIGURE 3:**
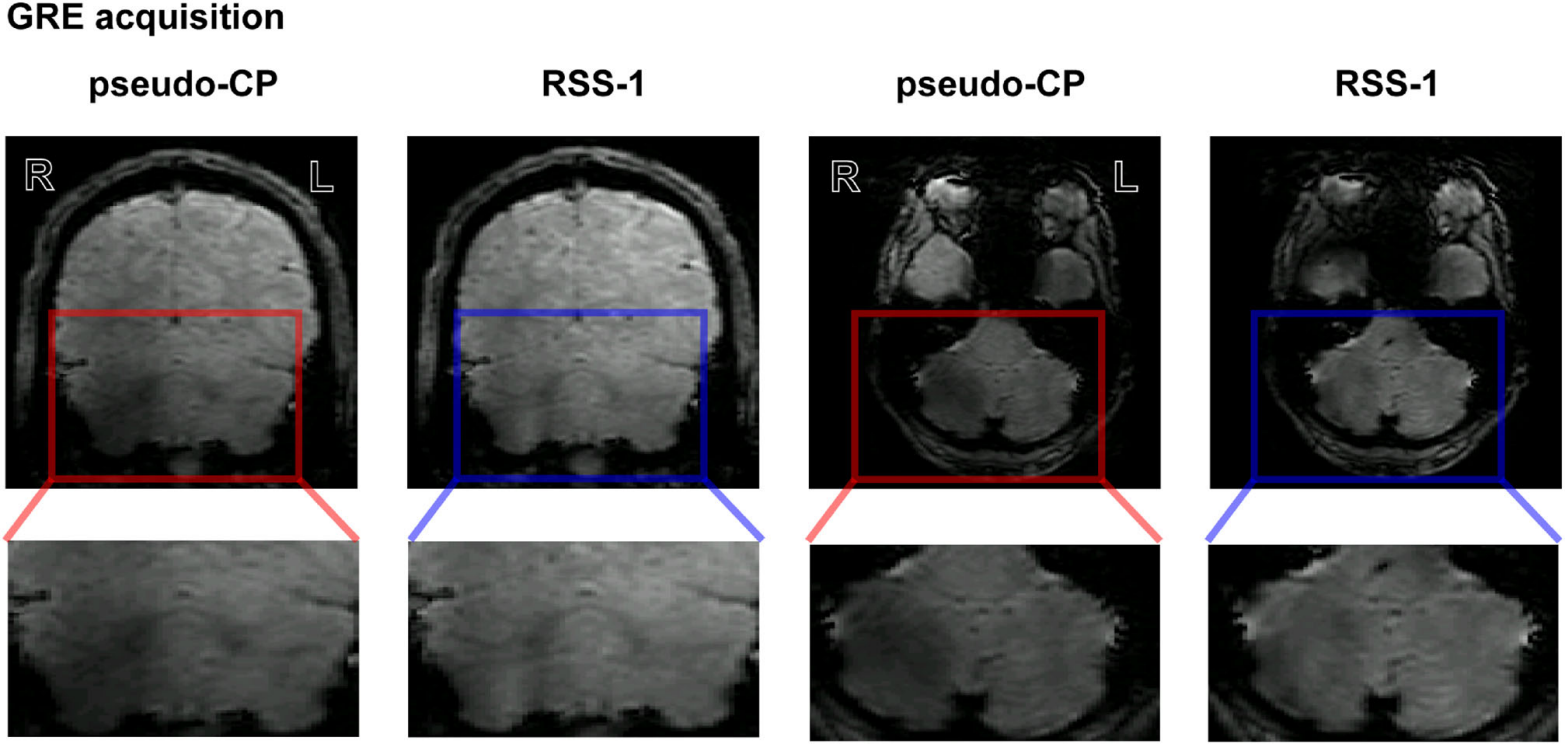
An example of the GRE acquisitions (group 3) using the pseudo‐CP mode and RSS‐1 (bottom row: cropped view). Presented for a coronal (left) and axial (right) orientation. Note the increase in signal intensity in the cerebellar ROI as well as a more homogeneous signal distribution between the left and right hemisphere in both acquisitions. GRE, gradient echo; pseudo‐CP, pseudo‐Circularly Polarized.

**FIGURE 4:**
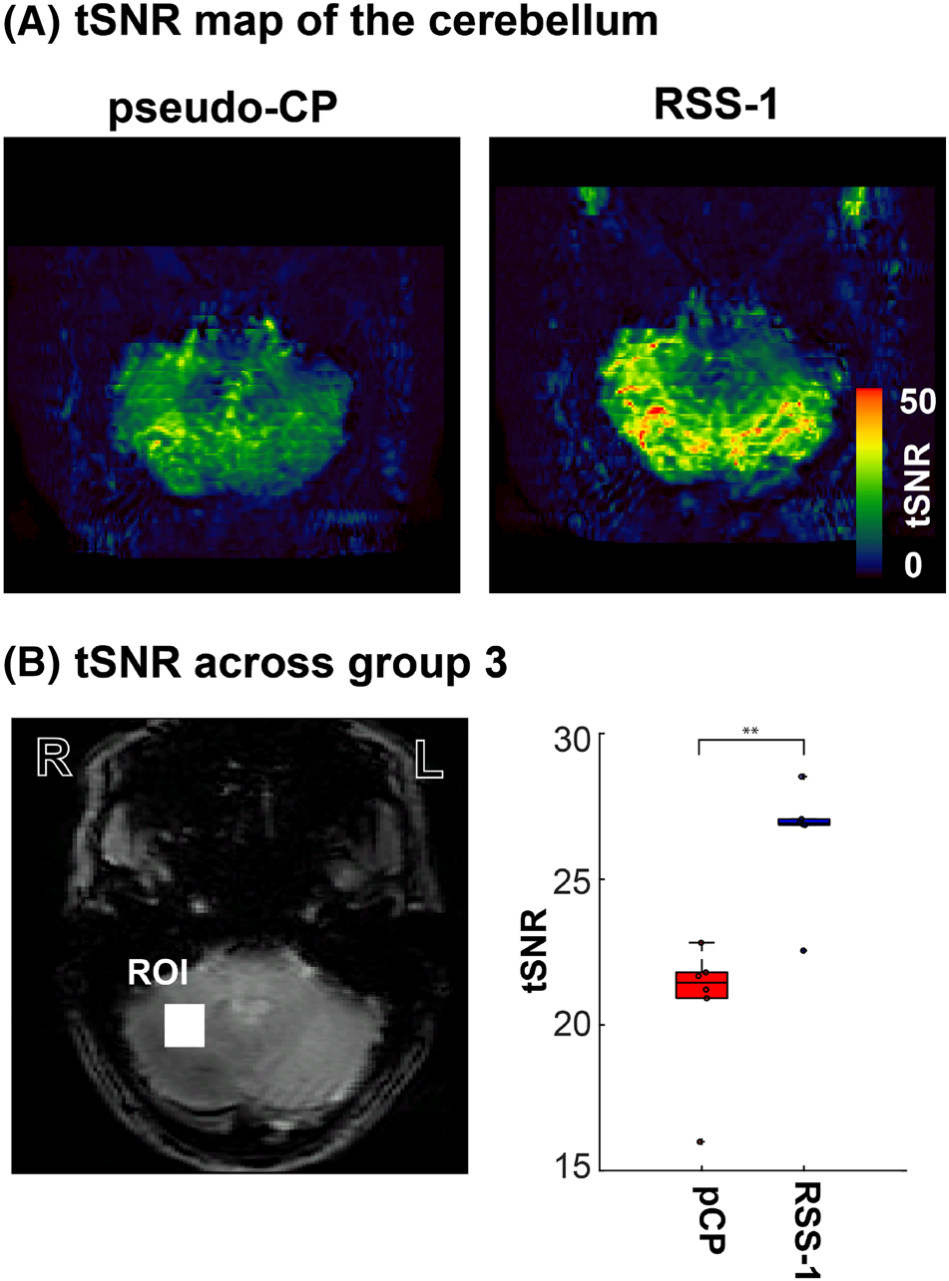
(A) An axial view of the calculated tSNR image for the pseudo‐CP mode and RSS‐1. Visually, the overall increase in tSNR in the cerebellar ROI in the RSS‐1 image is obvious. (B) tSNR in a cerebellar ROI (indicated by the white box in the reference image) across participants in group 3 for the pseudo‐CP mode (red) and RSS‐1 (blue). ** p<0.01.

Our simulation of the group‐optimized RSS‐1 showed significant increases (paired t‐test, p<0.001,d=−3.48) in the nominal FA percentages in the cerebellum compared to the pseudo‐CP mode, by 45%, thus improving $B_{1}^{+}$ yield (Figure 2C,D, Supporting Information Table 5, CP‐mode: red and group optimized: blue). This improvement was not significantly different to the improvements found with the personally optimized RSS‐1 (paired t‐test, p=0.25,d=−0.61; Figure 2C,D, Supporting Information Table 5). To illustrate universality across participants, FA percentages were also simulated for group 2 (Figure 2D, Supporting Information Table 5). Results from group 2 showed a similar distribution to that presented in group 1. Namely, a significantly higher FA in the cerebellum for RSS‐1 compared to pseudo‐CP mode (paired t‐test, p=0.001,d=−3.48). Our scanning results (group 3) confirmed this result, greatly increasing the nominal FA percentage in the cerebellum using RSS‐1 compared to the pseudo‐CP mode (Figure 2E and Supporting Information Table 5). This confirms that RSS‐1 can be generalized to participants outside the design group. The increase in nominal FA percentage using RSS‐1 (DREAM $B_{1}^{+}$; Figure 2B) resulted in visually improved signal in the cerebellum in both GRE (Figure 3) and 3D EPI (Supporting Information Figure 4 acquisitions). Specifically, signal intensity inhomogeneities between the left and right cerebellar hemispheres (as visible in the pseudo‐CP acquisition, Figure 3) were reduced successfully using RSS‐1. In addition, mean tSNR was significantly higher for all participants (paired *t*‐test, p<0.001,d=−2.6) using RSS‐1 (mean = 26.5, σ=2.02) compared to the pseudo‐CP acquisition (mean = 20.7, σ=2.42) in a cerebellar ROI (Figure 4B, Supporting Information Table 4).

### 3.2 Regional shim setting 2

**FIGURE 5:**
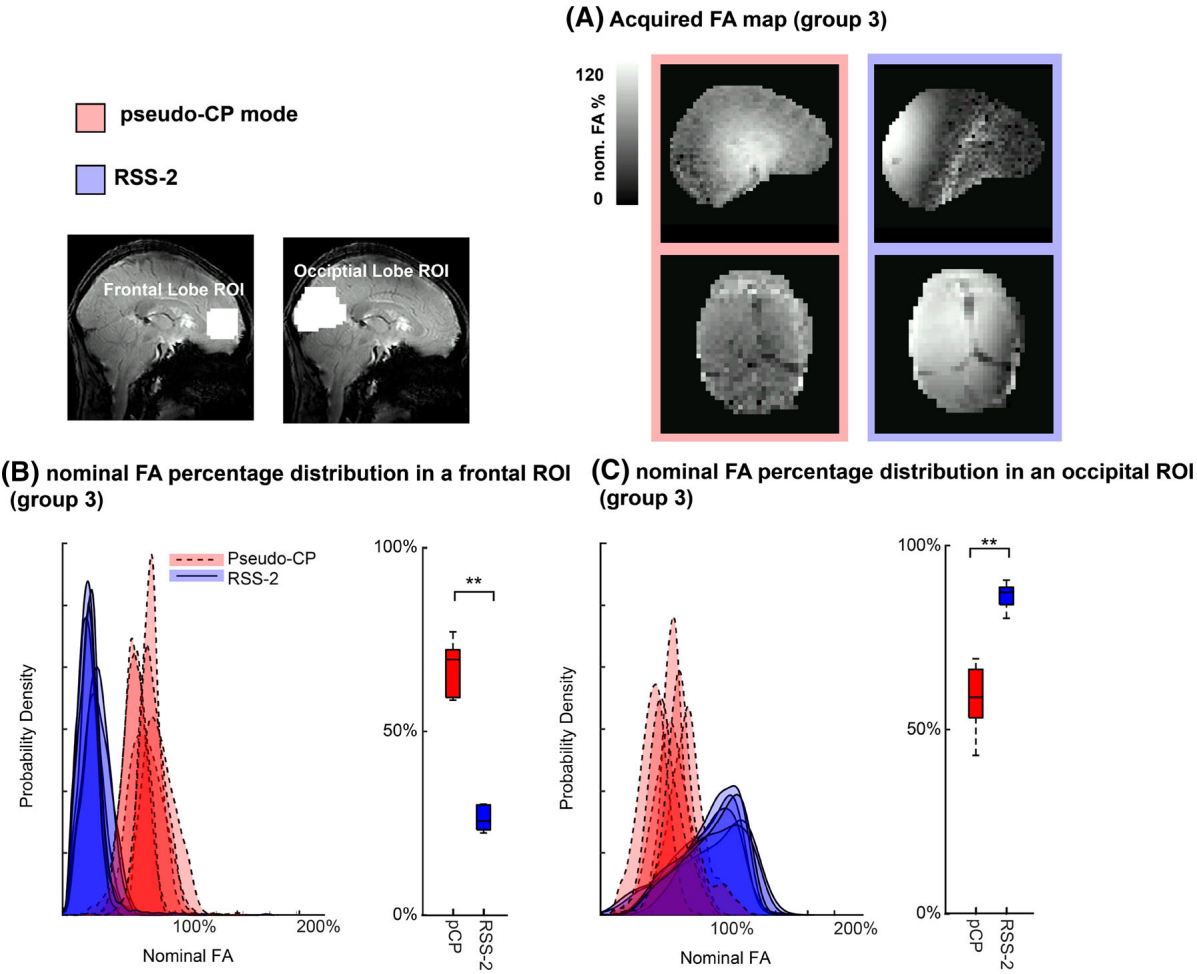
(A) An example flip angle map from a participant in group 3. (B) The acquired nominal flip angle percentage distribution in the occipital lobe for the pseudo‐CP mode (red) and RSS‐2 (blue). (C) The acquired nominal flip angle percentage distribution in the frontal lobe for the pseudo‐CP mode (red) and RSS‐2 (blue). Note the reduced flip angles across participants in the frontal lobe ROI using RSS‐2. ** p<0.01, FA, flip angle.

**FIGURE 6:**
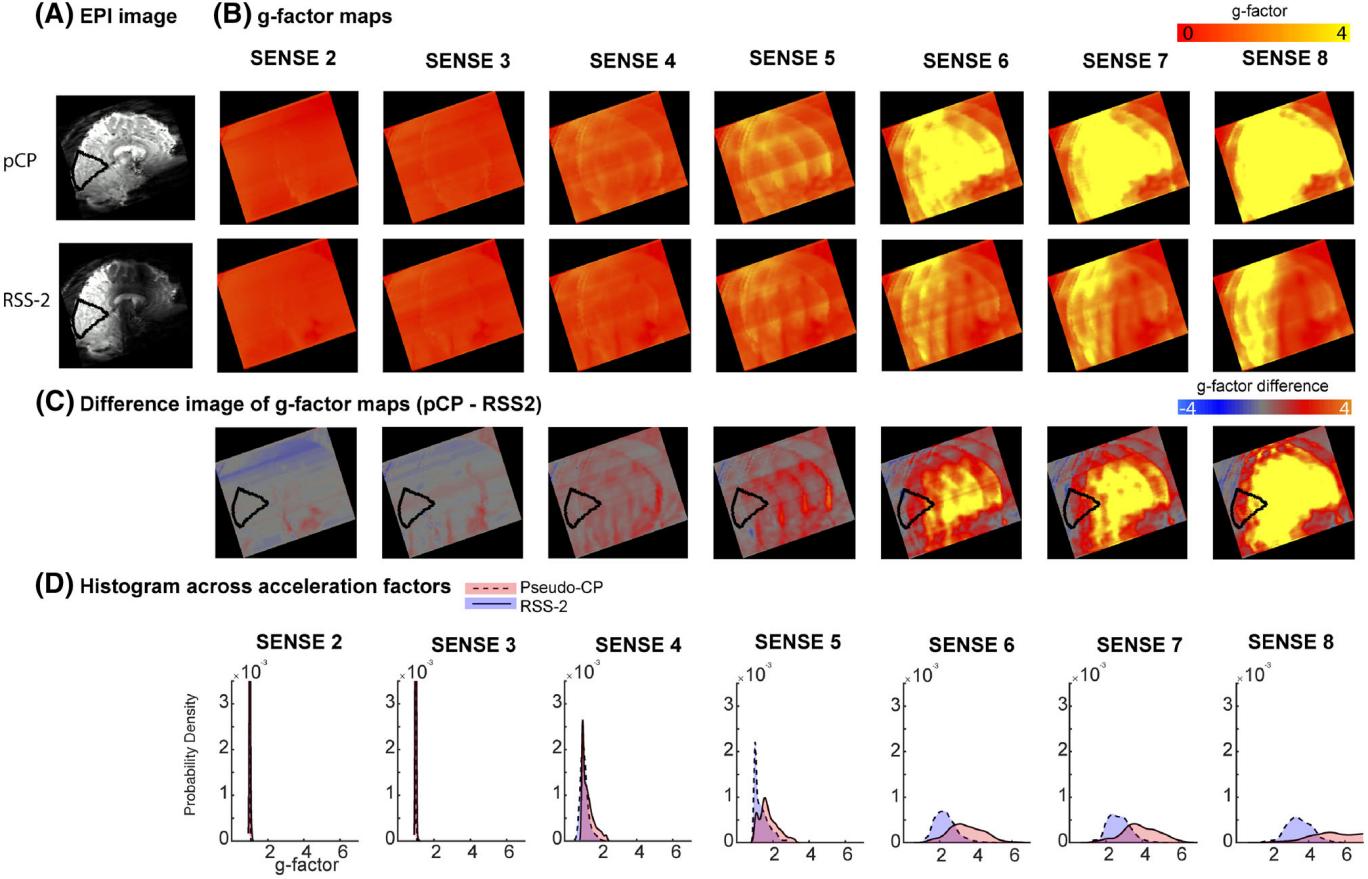
(A) EPI image acquired for acceleration factor 1 (SENSE 1) in pseudo‐CP mode (top) and RSS‐2 (bottom). (B) G‐factor maps for a single participant for different acceleration factors (SENSE) in the PA direction. Note how the g‐factors remain lower at higher acceleration factors for the RSS‐2 shim setting compared to the pCP mode. (C) The histogram plot of the g‐factor maps in a occipital lobe ROI. (D) Histograms of the g‐factor values in the occipital lobe ROI (indicated in C) across SENSE factors of 2‐8.

**FIGURE 7:**
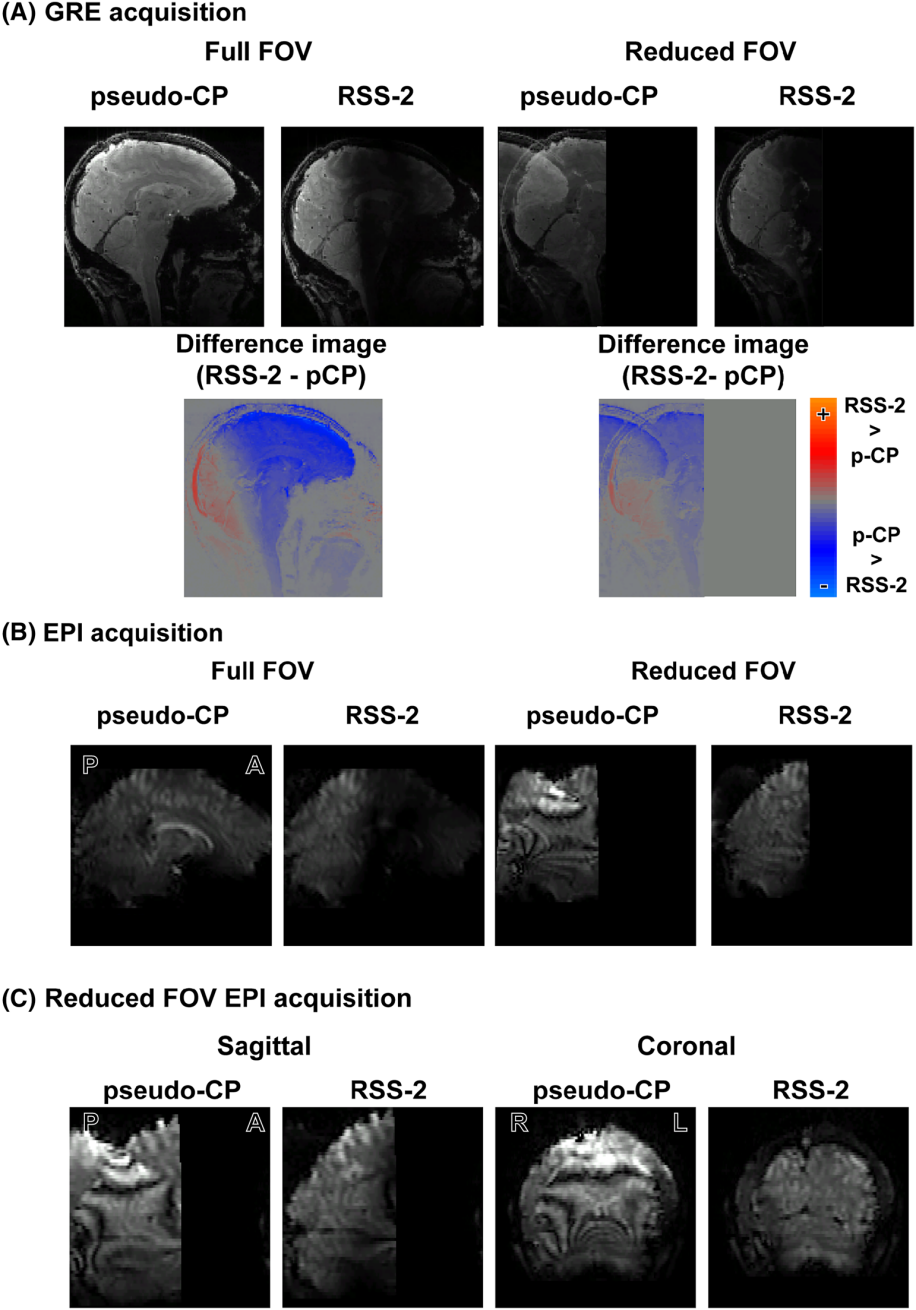
(A) An example of GRE images with SoS coil combination using RSS‐2 and the pseudo‐CP mode for the reduced FOV and full FOV acquisition (top). The difference image between RSS‐2 and pseudo‐CP images (bottom). Note the fold‐over into the back of the brain present for the cropped pseudo‐CP image but not for the cropped RSS‐2 image. (B) An example of the EPI acquisition image for the CP mode and using RSS‐2 for a reduced FOV and full FOV. (C) A zoomed‐in view of the fold‐over in the back of the brain using the reduced FOV acquisition presented for a sagittal (left) and axial (right) slice.

Having demonstrated that RSS‐1 calculated over group 1 can be generalized to participants outside the design group, we explored if regional generalized shim settings could be used to selectively attenuate as well increase the $B_{1}^{+}$ field in certain ROIs. The group‐optimized RSS‐2 significantly attenuated nominal FA percentage in a large region of the frontal cortex (Figure 5B, Supporting Information Table 5), while also significantly increasing nominal FA percentage in the occipital lobe compared to the pseudo‐CP acquisition (paired t‐test, p<0.001,d=−3.9; Figure 5C, Supporting Information Table 5). To explore the effect of signal attenuation in a second target region on undersampling performance, we compared vendor‐generated g‐factor maps (Figure 6B) obtained from scans with RSS‐2 or pseudo‐CP mode and varying undersampling factors. Across acceleration factors, g‐factors were lower for the RSS‐2 shim setting compared to the pseudo‐CP mode in a occipital lobe ROI (Figure 6C, Supporting Information Figure 3). Visually, the signal intensity in the frontal lobe in both GRE (Figure 7A) and 3D EPI (Figure 7B) acquisitions was effectively attenuated using RSS‐2. To demonstrate an implementation and the use in practice of RSS‐2, we halved the FOV to generate large fold‐over artifacts: for all participants, fold‐over and ripples into the back of the brain were visible in the reduced FOV pseudo‐CP GRE and 3D EPI acquisitions (Figure 7A,C). Visually, these artifacts were greatly decreased using group optimized RSS‐2 with the same reduced FOV (Figure 7B, Supporting Information Figure 4).

**FIGURE 8:**
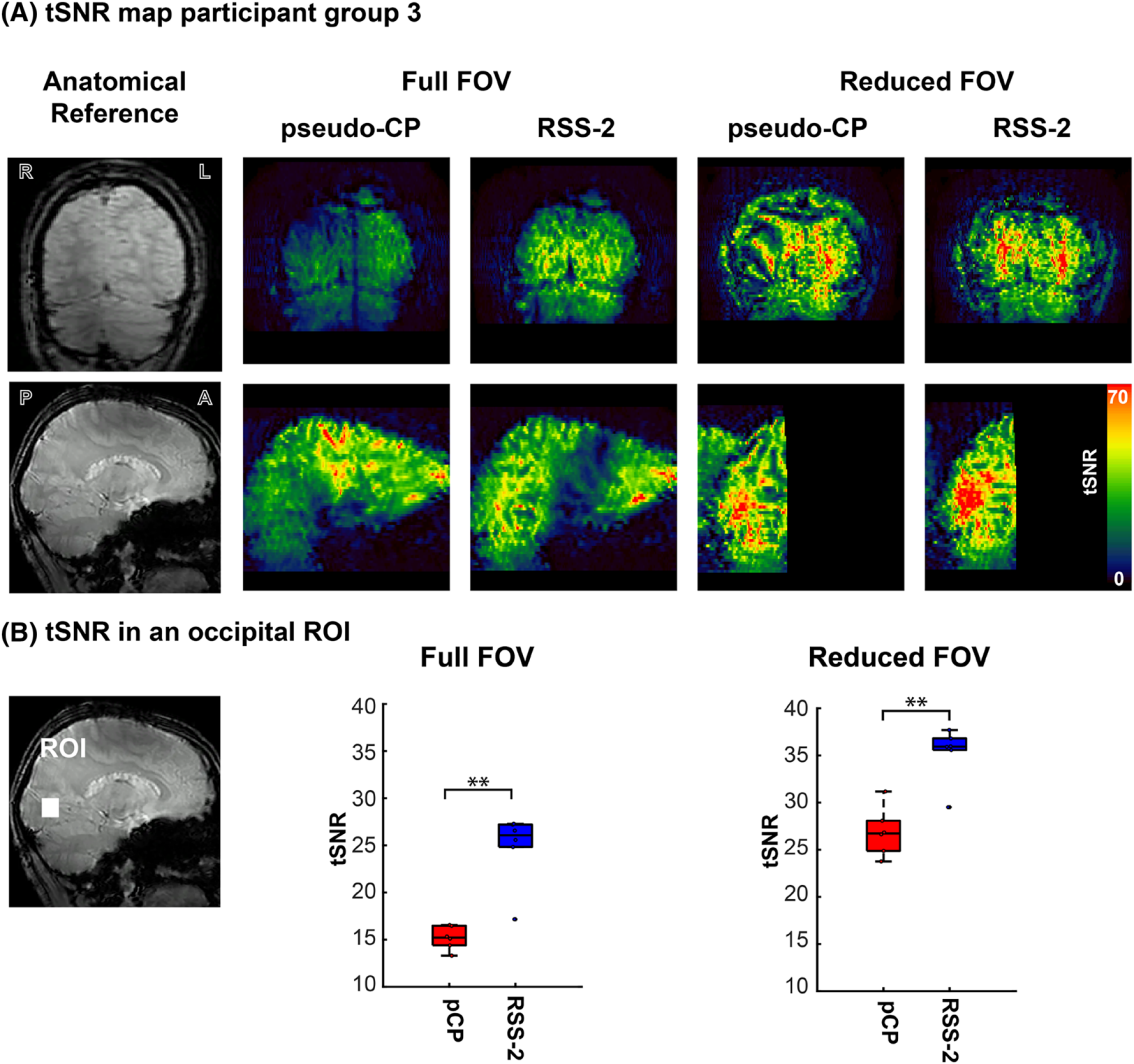
(A) A coronal (top) and sagittal (bottom) slice of the tSNR map for the pseudo‐CP mode (pCP) and RSS‐2. These are presented for both the reduced and full FOV. (B) Bar plots of tSNR across participants in group 3 in an occipital lobe ROI (indicated by the white box in the reference image) for the pseudo‐CP shim (red) and RSS‐2 (blue) and for full (left) and reduced FOV (right). ** p<0.01.

**FIGURE 9:**
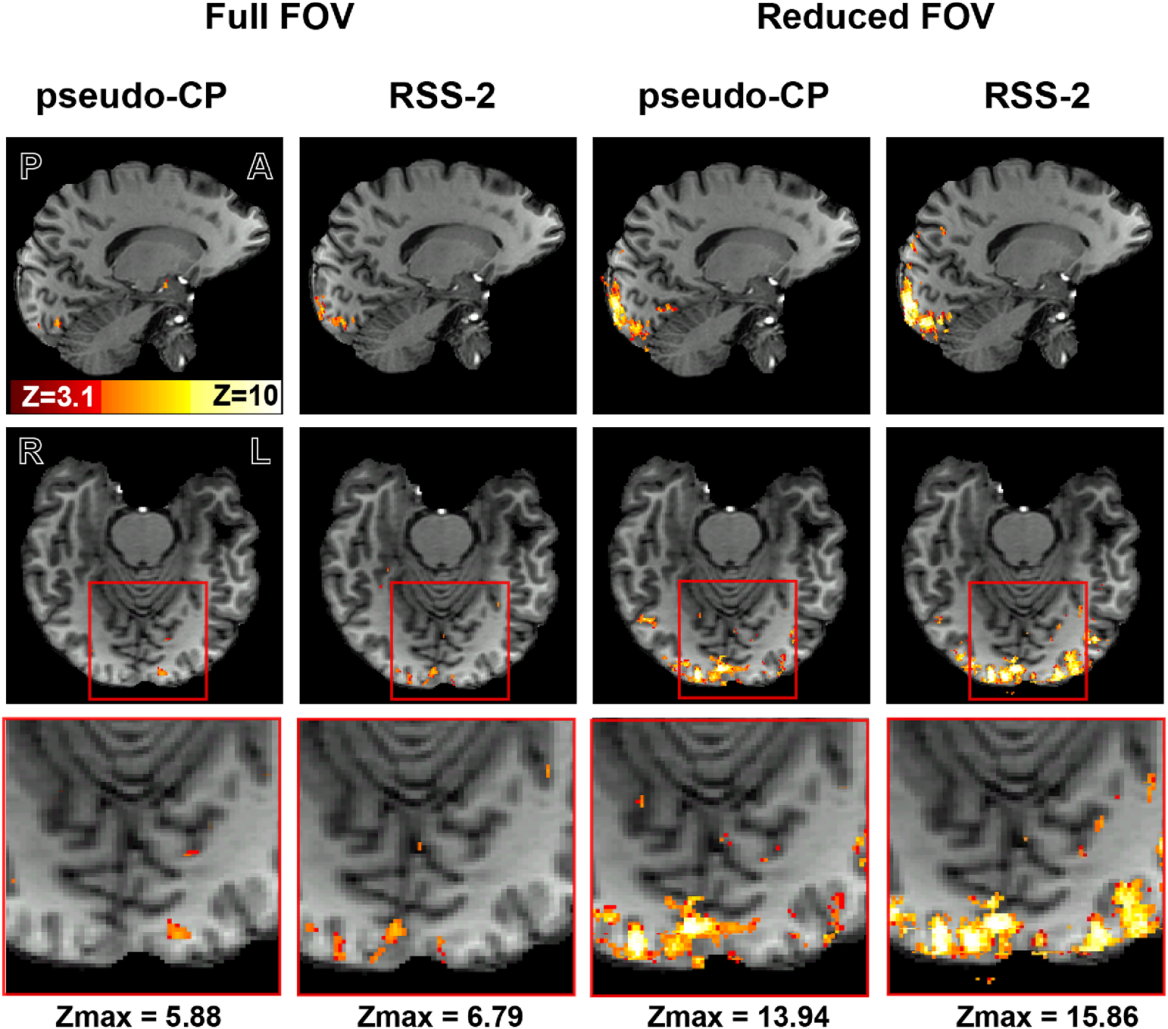
(A) The resulting z scores (z>3.1) for the pseudo‐CP mode and the attenuating shimmed acquisition. Results are presented for both the full FOV (left) and reduced FOV (right), overlaid onto an anatomical reference image (top row: sagittal; middle row: axial; bottom row: zoomed‐in axial view). The maximum z score for each acquisition is written in text below.

tSNR was higher in the occipital lobe using RSS‐2 for both the full and reduced FOVs (Full FOV: mean = 25, σ = 3.9, reduced FOV: mean = 35, σ = 3.0) compared to the pseudo‐CP acquisitions (Full FOV: Mean = 15, σ = 3.9, Reduced FOV: Mean = 27, σ = 2.6) (Figure 8B, Supporting Information Table 4). Reduced FOV acquisitions had higher tSNR in the occipital lobe compared to full FOV acquisitions (Figure 8B, Supporting Information Table 4). This difference might be explained by the increased physiological noise sensitivity in 3D EPI with increasing numbers of segments.^48^ The higher number segments is reflected in the longer volume acquisition times for the full FOV acquisitions. For one participant, we acquired 3D EPI acquisitions during a flickering checkerboard in the occipital lobe using RSS‐2 and pseudo‐CP mode and with both full and reduced FOVs. For all configurations, significant (p<0.05) BOLD responses were found in the occipital lobe during the flickering checkerboard task for both RSS‐2 and pseudo‐CP acquisitions. Because of the shorter volume acquisition time, double the number of volumes (*n*_vols_ = 90) was acquired using the reduced FOV acquisition compared to the full FOV acquisition (*n*_vols_ = 45) during the checkerboard task (6×30 s OFF 30 s ON) (Figure 8). Clusters (z>3.1) were larger using the RSS‐2/reduced FOV acquisitions (Figure 9). Maximum *z* scores were higher using RSS‐2 compared to the pseudo‐CP acquisitions (Figure 9).

## 4 DISCUSSION

$B_{1}^{+}$ field inhomogeneity remains a challenge in MR imaging at field strengths above 3 T.^9^, ^49^ Several pTx‐based approaches have been suggested to increase and homogenize the $B_{1}^{+}$ field in regions of interest. These approaches frequently require time‐consuming personalized calibrations, complex RF waveforms or they do not translate across the different sequences that may be used in a single scan session.^17^, ^23^, ^28^, ^29^, ^35^ Neuroscientific research at higher field strengths (>3T) frequently aims to investigate smaller areas of the brain with high specificity.^1^, ^8^, ^50^ To obtain high‐resolution images, it is crucial that sufficient SNR can be achieved. In contrast, signal coming from outside the investigated area can be a hindrance, necessitating longer (volume) acquisition times or causing artifacts driven by processes such as breathing and eye movements. These artifacts can interact and thereby be amplified by acquisition parameters such as undersampling factors.^51^ We examined if phase‐offset $B_{1}^{+}$ shims can generalize across participants when aiming to improve signal behavior in small brain regions typical for high‐resolution neuroimaging. We examined this for two different group‐optimized regional shim sets with a standard 8Tx/32Rx whole‐head coil and the water‐proton RF wavelength at 7 T. We demonstrate that we can calculate group phase offsets without significant performance loss compared to an individual local $B_{1}^{+}$ optimization. We further demonstrate that our group regional shim setting yields robust results when applied to unseen individuals outside the design group.

Currently, one of the biggest hurdles in the popularization of pTx shimming methods is ease of use. Our study demonstrates a simple approach that can be used without any input from the end‐user and can be generalized across sequences, for instance, in slab‐selective, slice‐selective, or whole‐brain sequences. Our signal improvements in brain imaging agree with changes observed in spectroscopic^35^ and spinal cord imaging studies^34^ employing group optimized $B_{1}^{+}$ shims. The simplicity of static $B_{1}^{+}$ shimming approaches makes them particularly attractive for neuroscientific and clinical applications where scan time is limited. Depending on the coil geometry, they can be generalized across different brain regions (e.g., the current geometry of the Nova transmit coil allows for some flexibility in the axial plane but not so much in the inferior–superior direction^24^). Note that static $B_{1}^{+}$ shims are not dependent on one vendor but can be readily applied in different sites. Acquisitions in field strengths above 7 T may particularly benefit from the even shorter RF wavelength that provides additional degrees of freedom.^52^ Similarly, coils with more transmit elements can also provide more degrees of freedom. In addition, if real time SAR supervision could be performed, relaxation of the power limits might be feasible, for an implementation to high flip angle pulses.

We further demonstrated that RSS‐2 can reduce signal contributions from regions‐of‐no‐interest while still increasing signal where it matters, without input from the user. In MRI research, there is frequently a need to alter the excitation profile of certain regions with respect to the imaging target, typically at the cost of either higher SAR (saturation bands) or complex dynamic‐pTx waveforms.^23^, ^31^ There are also several neuroscientific needs that have not been met due to increased acquisition time or complexity. For example, eye movements can alias into the occipital lobe when using undersampling factors^53^ or areas of high susceptibility can create dynamic within‐slice dephasing and distortions. We demonstrated that RSS‐2 successfully reduced signal contributions from areas with large vessels and the eyes as well as fold over artifacts that occurred when reducing the FOV of the acquisition. Additionally, RSS‐2 resulted in no effective receive signal in the receive channels near the frontal lobe areas, this resulted in less fold‐over and thereby higher acceleration factors could be used in the image acquisition. We were able to do this while at the same time improving tSNR in the occipital lobe (increased nominal FA percentage and tSNR). Note that in practice, our approach cannot completely remove signal over large regions: approximately 20% of the nominal flip angle remained. We therefore do not suggest universal destructive shims as an alternative for zoomed acquisitions with our particular combination of imaging parameters (transmit coil, FOV, and field strength). However, because our method consists of a simple static shim, similar to pCP mode, it is straightforward to simulate SAR and offer it as a functionality from the vendor. This is in contrast to individual or sequence dependent pTx approaches, where these simulations would be less practical. Further optimization, particularly with more complex coil designs or in combination with other approaches (saturation bands, local shims), may also be possible.

## 5 CONCLUSIONS

$B_{1}^{+}$ field inhomogeneity continues to be a challenge at higher field strengths (>3T), severely affecting signal quality in regions such as the cerebellum. Here, we explore if group‐optimized shims can provide a simple method to improve $B_{1}^{+}$ behavior in an area of interest while removing any burden to the user. We show that such approaches can both increase $B_{1}^{+}$ in specific regions of (neuroscientific) interest and locally reduce $B_{1}^{+}$ in a targeted manner, for example, to decrease artifacts, reduce the FOV, or facilitate higher acceleration factors.

## FUNDING INFORMATION

This work was supported by NWO TTW VIDI VI.Vidi.198.016, NWO Aspasia 015.014.038, NWO OCENW.XS22.4.007, and Amsterdam Brain and Cognition T0922 grants.

## Supporting information


**Data S1.** Supporting Information.

## Data Availability

The data that support the findings of this study are available from the corresponding author upon reasonable request.

## ENDNOTES

^1^

**Abbreviations:** pCP‐mode, Pseudo‐Circularly‐Polarized‐Mode: A close to circularly polarized mode achieved by B1‐shimming over the entire brain of a separate group of volunteers.^44^

^2^
Note that the ROI should be sufficiently large to avoid obtaining a low SD and high mean.
