# Supplementary material for: Easy‐to‐use B1+ shims for human brain imaging at 7 T
Source: Magn Reson Med. 2025 Jun 24;94(5):2010–22. doi: 10.1002/mrm.30617 (PMC12393206; doi:10.1002/mrm.30617)
Supplement: Supplementary file 1 — Data S1. Supporting Information. [file MRM-94-2010-s001.pdf]

SUPPLEMENTARY MATERIALS

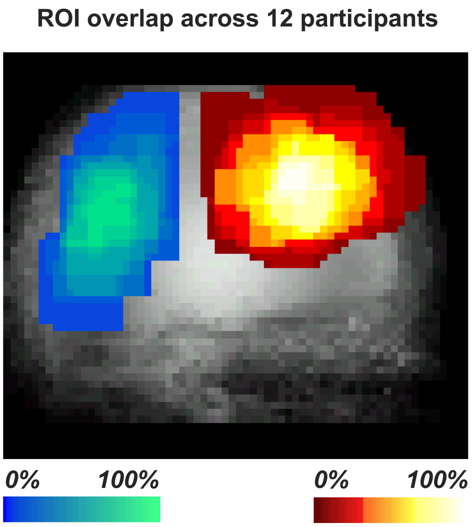

Figure S1 The average B1 map (greyscale) overlaid with the average occipital lobe ROI (blue/left) and the average frontal lobe ROI (red/right). (100% = all 12 participants overlap)

Table S1 The volume (cm^3) for each ROI

**Participant**	**Frontal ROI**	**Occipital ROI**
	cm^3	cm^3
1	125.7	65.9
2	111.3	103.0
3	104.8	98.2
4	159.5	130.6
5	145.1	82.4
6	136.0	76.3
7	199.2	82.7
8	147.6	88.0
9	129.0	87.2
10	114.5	102.2
11	137.3	112.9
12	99.5	119.2
**Mean**	132.0	94.6
**St Dev**	28.0	18.2

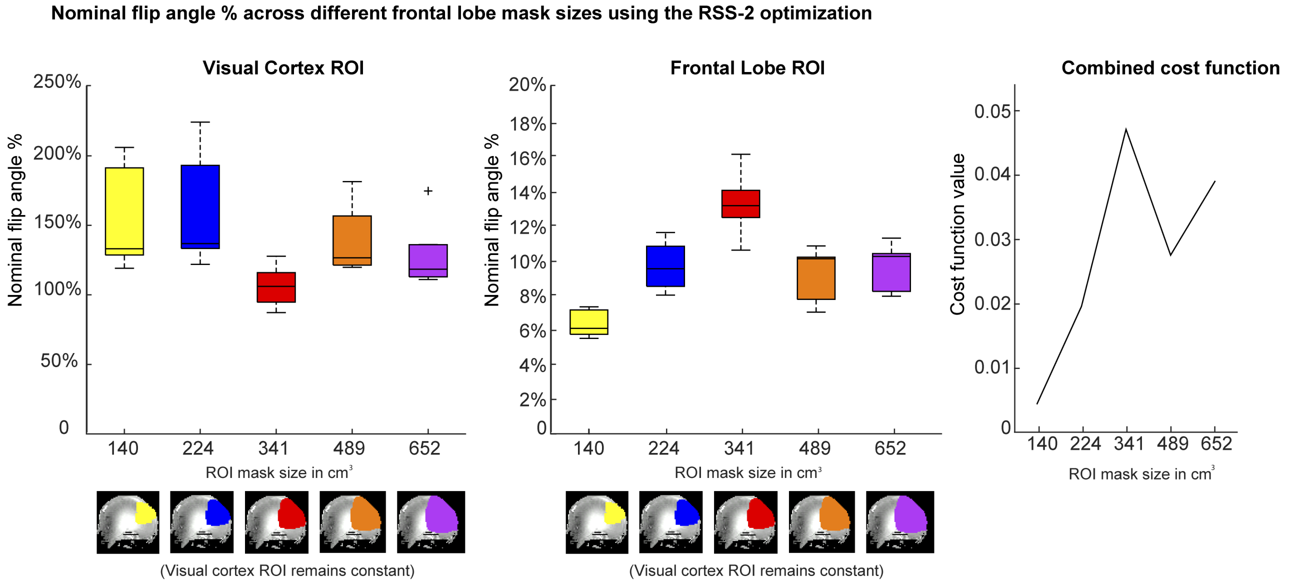

Figure S2 The nominal flip angle percentage in the occipital lobe ROI and frontal lobe when adjusting the size of the frontal lobe ROI in the RSS-2 cost function (i.e equations 4 and 5 in the manuscript). The occipital lobe ROI remained constant in these simulations. The horizontal axes shows the average frontal lobe mask size that was used for the optimisation. Where $140cm^3$ is the mask size as used in the manuscript in RSS-2 and the other ROIs are the initial mask dilated by 2,4,6 and 8 voxels. In the occipital lobe ROI flip angles are all above 100%, but they were highest using the original mask and the $224cm^3$ mask. The mean of the combined cost function (equation 5 in the manuscript) for each ROI is presented on the right. Note the lower values for the cost function for smaller ROIs

Table S2 Nominal % Flip Angle in the occipital lobe ROI , the ROI in the first row is the ROI size used in the frontal lobe for the optimization of RSS-2

Subject	ROI=140cm^3	ROI=224cm^3	ROI=341cm^3	ROI=489cm^3	ROI=652cm^3
1	165.00	179.35	107.63	152.33	135.65
2	130.70	136.59	106.08	121.76	111.09
3	200.01	197.63	118.85	181.39	174.66
4	133.14	136.90	93.38	126.70	118.49
5	119.15	121.96	98.92	119.79	114.88
6	127.98	132.38	87.31	121.26	112.37
7	205.96	224.23	127.76	158.22	136.21
Mean ± SD	154.71 ± 33.91	175.69 ± 32.67	105.42 ± 13.39	140.49 ± 22.45	129.05 ± 21.51

Table S3 Nominal % Flip Angle in the Frontal Lobe ROI , the ROI in the first row is the ROI size used in the frontal lobe for the optimization of RSS-2

Subject	ROI=140cm^3	ROI=224cm^3	ROI=341cm^3	ROI=489cm^3	ROI=652cm^3
1	7.27	9.58	14.31	10.25	10.47
2	5.95	10.14	13.55	9.64	8.67
3	7.37	11.14	13.20	10.25	10.43
4	6.12	11.68	13.24	10.16	10.30
5	5.54	8.78	12.33	7.06	7.99
6	5.71	8.48	10.65	7.19	8.13
7	6.93	8.05	16.21	10.90	11.35
Mean ± SD	6.27 ± 0.69	9.98 ± 1.36	13.64 ± 1.80	9.64 ± 1.39	9.76 ± 1.39

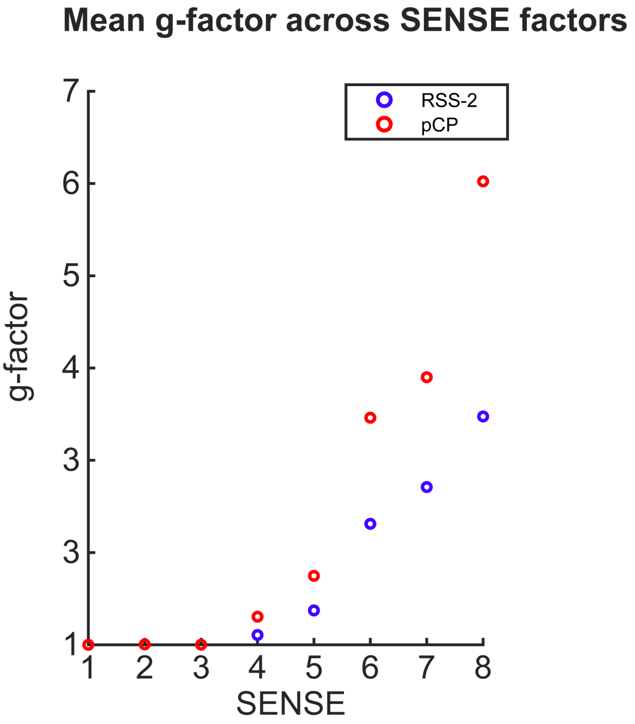

Figure S3 The mean g-factor across SENSE values in an occipital lobe ROI for the pseudo-CP shim compared to RSS-2.

Table S4 tSNR across different shim settings and ROIs.

	**ROI**	Pseudo-CP Mean (sd)	Group Mean (sd)	**Paired t-test** pCP - Group p	d
**RSS-1**					
Group 3	Cerebellum	20.74 (2.42)	26.47 (2.02)	p<0.001**	-2.57
**RSS-2 Full FOV**					
Group 3	Occipital Lobe	15.19 (1.24)	24.77 (3.85)	p<0.001**	-3.49
**RSS-2 Reduced FOV**					
Group 3	Occipital Lobe	26.89 (2.60)	35.25 (2.91)	p<0.001**	-3.03

p values are presented for a paired t-test d=Cohen's d, Group=Group optimized.

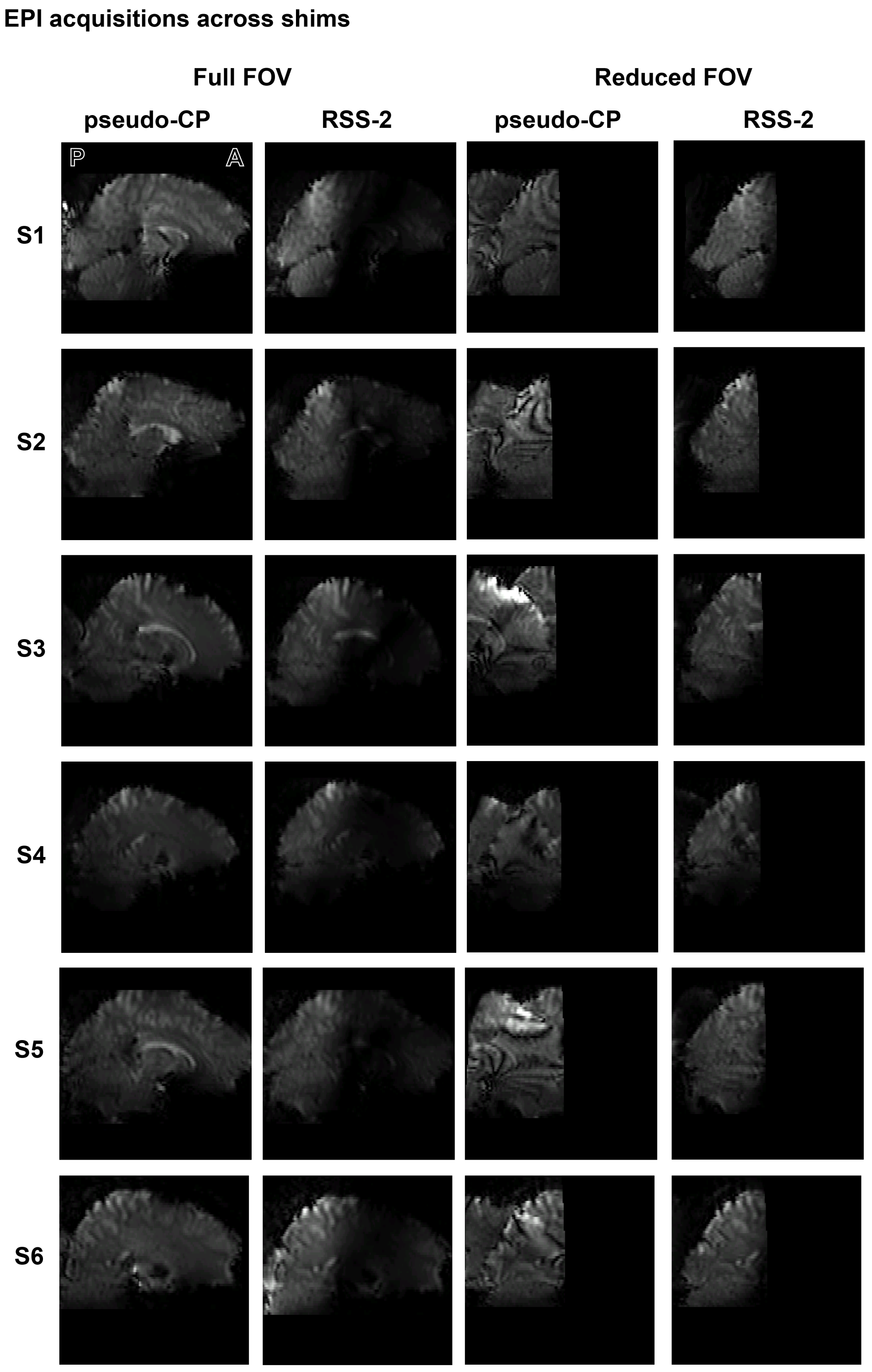

Figure S4 EPI acquisitions across shim settings and fields of view.

Table S5 Table 2: Percentage of nominal flip angle across regional shim settings .

	ROI	Pseudo CP	Group	Personalized	Paired t-test					
					Pseudo CP-group		Group-Personalized		Pseudo CP -Personalized	
		Mean (sd)	Mean (sd)	Mean (sd)	p-value	d	p-value	d	p-value	d
**RSS-1**										
Group 1	Cerebellum	57.95 (6.62)	105.60 (20.41)	120.44 (59.61)	<0.001**	-3.21	0.437	-0.33	0.027*	-1.5
Group 2	Cerebellum	54.17 (9.41)	99.41 (15.82)	106.57 (5.21)	<0.001**	-3.48	0.249	-0.61	<0.001**	-6.89
Group 3	Cerebellum	54.29 (6.94)	80.70 (9.47)		0.005**	-3.18				
**RSS-2**										
Group 3	Frontal Lobe	67.59 (7.45)	26.13 (3.36)		<0.001**	7.17				
Group 3	Occipital Lobe	58.34 (9.46)	86.54 (3.70)		0.002**	-3.93				

Simulated and measured flip angle percentages of the nominal value across different B1+ shims and ROIs. P values are presented for a paired t-test. d=Cohens d, * indicates $p<0.05$, ** indicates $p<0.001$. The large standard deviation resulting from the personalised shim in Group 1 is due to a single participant with a very high simulated nominal percent flip angle. The standard deviation is lower for the group optimized offsets.
